# Editorial: Neuroscience, learning, and educational psychology

**DOI:** 10.3389/fpsyg.2022.928054

**Published:** 2022-07-21

**Authors:** María Jesús Luque-Rojas, Eduardo Blanco Calvo, María Teresa Martín-Aragoneses

**Affiliations:** ^1^Research Methods and Diagnosis in Education, Faculty of Education, University of Malaga, Málaga, Spain; ^2^Psychobiology and Behavioural Science Methods, Faculty of Psychology, University of Malaga, Málaga, Spain; ^3^Research Methods and Diagnosis in Education, Faculty of Education, National University of Distance Education, Madrid, Spain

**Keywords:** neuroscience, education, learning, psychology, research and development, brain

Studying neuroscience within the educational context is a necessary effort and, in some cases, a mandatory one. However, an educational environment cannot be limited solely to the classroom; there is so much more, including, but not limited to, self-development, interactions, and relationships with the teacher. In this same line, it is essential to research studies that can provide us with rich, valid, and complete information from a neuropsychological perspective, particularly to the students within the context that defines them culturally, personally, familiar, and academically.

Advances in the contribution of neuroscience to education and personalization of learning could be the sentence that summarizes the main interest of publications in this Research Topic. The topic of Neuroscience and Education is already gaining attention as an emerging research field. Scientists are starting to discover that the relationships within the brain are strongly linked to improving abilities both within students and teachers. Despite limited research in Educational Neuroscience, it is a new and innovative area of study where we can find a large amount of development. These studies contribute significantly to discoveries and materials in this growing field.

Through this research, we want to delve deeper into the connection between Neuroscience and Education beyond just academic performance to avoid confusing classroom work with activities based on emotion, cognition, and motivation. Nowadays, we can see how the term neuroeducation is being used frequently without connecting it to the objective analysis of the brain and its multiple options in some educational contexts and schools. The current Research Topic collects 14 articles under world-leading authors from different specialization areas (Psychology, Neuroscience, Education, Pedagogy, and so on.). Articles published belong to four topics: Neuroscience, Education, Learning, and Educational Psychology (Table 1).

**Table 1:** Conditions of articles in the Research Topic.

	**Research methods**	**Journal**	**References**	**Data collection methods and tools**	**Study group**
1	Exp	Psychology	Liu, Wang et al.	Behavioral Skin conductance Heart rate	21–23
2	Exp	Psychology	Zhao et al.	Behavioral	15
3	Perspective	Education	Macrine and Fugate	Behavioral/Technologies	–
4	Systematic Review-Meta-analysis	Psychology	De-la-Peña and Luque-Rojas	Behavioral	18–25
5	Brief Research Report	Psychology	De-la-Peña et al.	Behavioral	17–47
6	Exp	Psychology	Poon et al.	Behavioral	8–10
7	Exp	Psychology	Alhossein	Behavioral	Teachers
8	Exp	Education	Luu-Thi et al.	Behavioral	Teenagers
9	Exp	Psychology	Wang et al.	Behavioral	23–35
10	Exp	Psychology	Aranda et al.	Behavioral	University students
11	Exp	Psychology	Bartolomé-Anguita et al.	EEG Behavioral	Young Adult
12	Hypothesis and theory article	Psychology	Liu et al.	Eye tracking Electroencephalography recording Behavioral	University students
13	Exp	Psychology	Shi and Qu	EEG Eye tracking Behavioral	Teenagers
14	Opinion	Psychology	Gola et al.	–	–

With this particular Research Topic, we would like to present the original studies of Neuroscience in Education to the educational science community on studying the brain and their answers to Comprehensive Education (Personal, Social, Cognitive, and Emotional Development).

Considering the neuroscientific studies, Gola et al. indicated that educational neuroscience research “that invests at different levels the theories and practices of education are not new.” However, we must focus on the different processes studied in this field, the biological perspective in education, and the relationship between cognitive processes (e.g., executive functions) and learning (e.g., stress and the difficulty of learning). Gola et al. also emphasized the use of neuroscientific techniques “into the psychological theory of educational constructs (such as reading).” In this sense, to measure the effectiveness of color-coding, Liu, Ma et al. used two neurophysiological tools: (1) eye-tracking, and (2) electroencephalography (EEG). Liu, Ma et al. showed that the color-coded design was more beneficial than the grayscale design (e.g., smaller pupil diameter), indicating changes in brain EEG activity (e.g., higher theta and alpha band power) and better learning performance. Moreover, Shi and Qu studied the effects of cognitive ability and self-control on comprehensive academic performance. They used several tools, including behavioral tests and neuroscientific tools, EEG, and eye-tracking. The results indicated a direct relationship between cognitive processes (memory, information processing, representation ability, logical reasoning ability, and thinking transformation ability) and self-control.

Similar to other studies on this Research Topic, the effects of collaborative learning were measured using psychophysiological techniques. Liu, Wang, et al. described the relationship between interpersonal physiological synchrony and collaborative learning activities while measuring electrodermal activity (EDA) and heart rate (HR) when students participated in lessons comparing synchrony between independent tasks and group discussion activities. Liu, Wang et al. indicated that high collaboration pairs gave significantly higher EDA and HR synchrony during the group discussions than low collaboration dyads. We can find another article (Bartolomé et al., 2022) that studied coaching as a human development tool using EEG and three experimental conditions: rumination®, directive (DC), and non-directive coaching (NDC). EEG indicated changes in alpha and theta frequencies in the right temporal region, and alpha, theta, and gamma in the right parietal region were measured in the NDC compared to R and DC conditions. The results were related to creativity and the development of human knowledge.

Along with the research of Liu, Ma, et al. and Shi and Qu, but under behavioral approaches and learning processes, Luu-Thi et al. demonstrated the link between mathematics anxiety and academic coping strategies, gender, grade, and career choices. Like Luu-Thi et al., de-la-Peña et al. studied attitudes toward mathematics and its relationships with other cognitive processes, like creativity and cognitive flexibility. The authors confirmed that details and cognitive flexibility were good predictors of a positive attitude toward mathematics. These results could have implications for educational practice in the planning of mathematics instruction in higher education, specifically referring to the work of future teachers.

The behavioral approaches and learning processes under cognitive processes permeate the other three articles. Firstly, Poon et al. showed that children with ADHD, who are predominantly inattentive subtypes and have reading difficulties, exhibited diverse cognitive profiles. Poon et al. observed that students with reading difficulties were related to verbal and visual-spatial working memory deficits; however, ADHD students, predominantly the inattentive subtype, were associated with behavioral working memory deficits. Second, Zhao et al. studied the relationship between self-esteem and academic engagement in an adolescent sample, moderated by other variables: academic self- efficacy and perceived social support. The authors established that when students felt more social support, they increased their academic self-efficacy in their academic engagement. Based on the findings, the authors emphasize the importance of adolescent self-esteem, academic self-efficacy, and perceived social support as crucial factors within educational and familiar contexts. In the third, Alhossein talked about the teachers' knowledge and the use of evidence-based practices (EBPs) for students with autism spectrum disorder (ASD). The author showed that knowledge and use of EBPs were closely related. Also, he investigated two other predictors of teachers' use of EBPs for students with ASD, gender, and professional development programs. On the other hand, Alhossein indicated that teachers' knowledge of EBPs for students with ASD could be considered a vital indicator of teachers' use of EBPs. Applying these kinds of programs and practices would offer high-quality professional development programs through this research.

We can link these findings to the perspective of Macrine and Fugate about the importance of embodied cognition and learning. The authors analyzed and discussed their model, Translational Learning Sciences Research. They were convinced thoroughly of the importance of this model to improve new research methods in the cognitive, educational, and psychological fields while increasing the use of tools and new learning strategies in the classroom. Similar to Macrine and Fugate and Wang et al. analyzed a model of the effectiveness of online instructional tasks. They indicated the same effectiveness of the online program on lower-performing students and higher- performing students.

In summary, the contributions of neuroscience to the educational context are essential both for teachers to know what the most effective way is to educate and for students to take advantage of the instructional environment efficiently. In this sense, we need more future research to apply the significant advantages of modern brain recording and neuroimaging. On the other hand, the techniques in teaching-learning situations in academic educational contexts are accompanied by behavioral, pedagogical, and educational studies. They all explain what or how we should develop a teaching style that fosters meaningful learning in students, with the goal of forming more intelligent, conscious, and committed thinkers to solve the current challenges of our society.

## Author contributions

ML-R and EB wrote themanuscript. All authors contributed to the article and approved the submitted version.

## Conflict of interest

The authors declare that the research was conducted in the absence of any commercial or financial relationships that could be construed as a potential conflict of interest.

## Publisher's note

All claims expressed in this article are solely those of the authors and do not necessarily represent those of their affiliated organizations, or those of the publisher, the editors and the reviewers. Any product that may be evaluated in this article, or claim that may be made by its manufacturer, is not guaranteed or endorsed by the publisher.

